# Impact of diabetes on COVID-19-related in-hospital mortality: a retrospective study from Northern Italy

**DOI:** 10.1007/s40618-020-01382-7

**Published:** 2020-08-10

**Authors:** S. Ciardullo, F. Zerbini, S. Perra, E. Muraca, R. Cannistraci, M. Lauriola, P. Grosso, G. Lattuada, G. Ippoliti, A. Mortara, G. Manzoni, G. Perseghin

**Affiliations:** 1 Department of Medicine and Surgery, Università degli Studi di Milano Bicocca, Milan, Italy; 2 Department of Medicine and Rehabilitation, Policlinico di Monza, Via Modigliani 10, 20900 Monza, MB Italy; 3 Department of Anesthesiology and Intensive Care, Policlinico di Monza, Monza, Italy; 4 Department of Clinical Cardiology, Policlinico di Monza, Monza, Italy

**Keywords:** Diabetes, COVID-19, Coronavirus, Mortality, SARS-CoV-2

## Abstract

**Purpose:**

The purpose of this study was to evaluate the impact of pre-existing diabetes on in-hospital mortality in patients admitted for Coronavirus Disease 2019 (COVID-19).

**Methods:**

This is a single center, retrospective study conducted at Policlinico di Monza hospital, located in the Lombardy region, Northern Italy. We reviewed medical records of 373 consecutive adult patients who were hospitalized with COVID-19 between February 22 and May 15, 2020. Data were collected on diabetes status, comorbid conditions and laboratory findings. Multivariable logistic regression was performed to evaluate the effect of diabetes on in-hospital mortality after adjustment for potential confounding variables.

**Results:**

Mean age of the patients was 72 ± 14 years (range 17–98), 244 (65.4%) were male and 69 (18.5%) had diabetes. The most common comorbid conditions were hypertension (237 [64.8%]), cardiovascular disease (140 [37.7%]) and malignant neoplasms (50 [13.6%]). In-hospital death occurred in 142 (38.0%) patients. In the multivariable model older age (Relative Risk [RR] 1.06 [1.04–1. 09] per year), diabetes (RR 1.56 [1.05–2.02]), chronic obstructive pulmonary disease (RR 1.82 [1.13–2.35]), higher values of lactic dehydrogenase and C-reactive protein were independently associated with in-hospital mortality.

**Conclusion:**

In this retrospective single-center study, diabetes was independently associated with a higher in-hospital mortality. More intensive surveillance of patients with this condition is to be warranted.

## Introduction

Coronavirus Disease 2019 (COVID-19), caused by severe acute respiratory syndrome coronavirus 2 (SARS-CoV-2) emerged in Wuhan in December 2019. The disease rapidly spread throughout the world and was announced as a global pandemic by the World Health Organization. Italy was among the first and most severely affected countries and by the time we are writing (end of May 2020) 225.549 cases and 30.332 deaths occurred, most of which in the Northern part of the country, with the Lombardy region accounting for more than a third of cases and half deaths (https://www.epicentro.iss.it).

Since the infection can have a broad range of severity going from an asymptomatic state, to symptoms similar to those of the common cold, to severe forms of interstitial pneumonia that need urgent medical attention, knowledge of risk factors for adverse clinical outcomes would be of great utility [1]. It soon became apparent that age and number of comorbidities were crucial to predict the need of intensive care unit (ICU) admission and death [2].

Diabetes is common among patients who die from COVID-19, with data from Italy showing a prevalence of approximately 30% [3]. Moreover, it is well known that patients with diabetes have higher risk of infections because of alterations in the immune response [4]. Nonetheless, it remains controversial whether diabetes could be considered an independent risk factor for greater severity of illness and death, with some studies showing a detrimental effect [5–7] and others a neutral influence [8–10], also depending on adjustment for confounding variables. Moreover, the vast majority of the published literature comes from China, and little is known in western countries and Italy in particular [11].

Therefore, the present study was conceived to obtain more conclusive information on the relationship between a history of diabetes and the risk of in-hospital mortality.

## Methods

### Study sample

This single center retrospective study was conducted at Policlinico di Monza hospital in Monza, a ~ 240-bed community based hospital designated to treat patients with COVID-19, located in the Lombardy region, Italy. We included all patients aged ≥ 18 years who were admitted from 22 February 2020 and experienced a definite outcome (either in-hospital death or discharge) as of 15 May 2020. These patients have not been reported before and represent a large spectrum of disease severity, including critically ill patients treated in the ICU.

COVID-19 cases were defined according to a positive result on real-time reverse transcriptase-polymerase chain reaction (RT-PCR) of nasopharyngeal or oropharyngeal swab specimens and/or clinically by the presence of typical signs and symptoms, exposure to known affected individuals and radiographic findings consistent with interstitial pneumonia.

The investigations were conducted in accordance with the current Declaration of Helsinki. The study was approved by the local ethical committee and the requirement for informed consent was waived because of the retrospective design and the ongoing public health emergency.

### Procedures and definitions

Using electronical medical records, we reviewed data related to demographic variables (age, gender, and occupational status), previous medical history and comorbidities and prescription medications. Date of admission, total hospital length of stay, presenting symptoms (including fever, cough, dyspnea, diarrhea and conjunctivitis), plasma and serum based biomarkers drawn within 48 h of hospital admission or transfer from different hospitals, and radiographic findings at presentation were also reviewed by a team of experienced clinicians. Routine laboratory examinations included a complete blood cell count, liver (aspartate aminotransferase, [AST] and alanine aminotransferase, [ALT]) and renal function testing, lactic dehydrogenase (LDH), creatine kinase and c-reactive protein (CRP).

Fever was defined as an axillary temperature ≥ 37.5 °C. Medical comorbidities of interest included diabetes, hypertension, previous cardiovascular disease (CVD), a composite of coronary artery disease, stable angina, stroke and transient ischemic attack, previous or active malignant neoplasia, chronic obstructive pulmonary disease (COPD), asthma, neurological conditions (such as dementia and parkinsonism), chronic kidney disease (CKD) and hematological disorders.

### Statistical analysis

Data are expressed as means ± standard deviation (SD) for continuous variables or as numbers and percentages for categorical variables. Analyses were performed using the SPSS software (version 24.0; SPSS, Chicago, IL). Normal distribution was assessed by the d’Agostino D-normality test [12] and skewed data were properly transformed before the analysis.

Categorical variables were compared by Pearson chi-square test. Independent sample *T*-test or Mann–Whitney test were used to compare groups. Levene’s test was performed to assess equality of variance. The percentage of missing values was equal to 9.12% and a listwise deletion approach was used to manage missing data.

Binomial logistic regression analysis was performed to ascertain the effects of several variables on the likelihood of mortality. Linearity of the continuous variables was assessed via the Box-Tidwell procedure and multi-collinearity was tested.

Given the number of deaths in our population, we included eleven independent variables in our model to avoid overfitting. Age and gender were included as males and older individuals were shown to have a higher mortality risk in previous studies [13]. Beside diabetes, we included other comorbidities that were significantly more common among deceased participants, such as hypertension, CVD, COPD and CKD. Finally, laboratory exams that were previously reported as predictive of severe disease or distributed unequally between discharged and deceased patients were included (platelet count, LDH, CRP and creatinine). Despite being associated with both diabetes and mortality, we did not include AST levels in the multivariate models as they were missing in a substantial number of patients.

Due to missing data, analysis was carried out in a subgroup of 339 subject from the 371 of the entire population.

The False Discovery Rate approach was used to control for multiple comparisons and the Benjamini and Hochberg procedure was assessed to minimize possible type I errors using a *q *value equal to 0.10 [14]; adjusted *p *values were reported.

A *p *value ≤ 0.05 was considered to be significant.

## Results

### Population characteristics

Of the 373 patients included in the study 129 were females (34.6%) and 244 were males (65.4%). Mean age was 72 ± 14 years (range 17–98) and only 51 individuals (13.7%) were younger than 55. 319 patients (85.5%) had a positive RT-PCR result and the remaining 54 (14.5%) tested negative and were diagnosed clinically. Apart from being significantly older than confirmed cases (76 vs 71 years, *p* = 0.12), patients with clinically diagnosed COVID-19 exhibited similar clinical features and comorbidities.

As shown in Table 1, most patients had comorbidities, the most common being hypertension (64.8%), followed by CVD (37.7%) and 69 patients had diabetes (prevalence of 18.5%). The most common presenting symptom was fever (82.6%), followed by dyspnea (71.3%) and cough (39.1%). Non-respiratory symptoms and signs, such as diarrhea and conjunctivitis were much less commonly reported. 58 patients were admitted to the ICU, of which 14 (24.1%) were transferred from other hospitals on mechanical ventilation.

**Table 1:** Demographic and laboratory features of patients with diagnosed COVID-19 segregated by mortality

	All patients	Discharged	Deceased	*p *value
*N* (female/male)	373 (129/244)	231 (146/85)	142 (44/98)	0.252
Age (years)	72 ± 14	68 ± 14	78 ± 10	< 0.001
≤ 55 (%)	13.7% (51)	19.9% (46)	3.5% (5)	< 0.001
56–75 (%)	37.8% (141)	44.2% (102)	27.5% (39)	
76–85 (%)	35.4% (132)	29.0% (67)	45.8% (65)	
> 85 (%)	13.1% (49)	6.9% (16)	23.2% (33)	
Intensive Care Unit (%)	15.5% (58)	6.5% (15)	30.3% (43)	< 0.001
Comorbidities				
Diabetes (%)	18.5% (69)	15.6% (36)	23.2% (33)	0.064
Hypertension (%)	64.8% (237)	56.8% (130)	78.1% (107)	< 0.001
CKD (%)	12.9% (48)	9.5% (22)	18.3% (26)	0.014
Tumors (%)	13.6% (50)	12.2% (28)	15.8% (22)	0.320
Cardiovascular diseases (%)	37.7% (140)	32.5% (75)	46.4% (65)	0.007
COPD (%)	10.6% (39)	6.5% (15)	17.4% (24)	0.001
Symptoms at admission				
Cough (%)	39.1% (146)	45.9% (106)	28.2% (40)	0.001
Dyspnea (%)	71.3% (266)	62.3% (144)	85.9% (122)	< 0.001
Fever (≥ 37.5 °C) (%)	82.6% (308)	82.3% (190)	83.1% (118)	0.834
Diarrhea (%)	4.8% (18)	6.1% (14)	2.8% (4)	0.159
Conjunctivitis (%)	0.8% (3)	0.4% (1)	1.4% (2)	0.306
Laboratory features at admission				
White blood cells (10^9^/l)	8.00 ± 4.02	7.72 ± 3.99	8.46 ± 4.05	0.019
Lymphocytes (10^9^/l)	1.05 ± 1.12	1.13 ± 0.81	0.91 ± 1.49	< 0.001
Neutrophils (10^9^/l)	6.33 ± 3.7	5.91 ± 3.75	7.02 ± 3.52	< 0.001
Hemoglobin (g/dl)	12.83 ± 2.04	12.89 ± 1.98	12.74 ± 2.14	0.382
Platelets (10^9^/l)	225 ± 107	242 ± 114	196 ± 87	< 0.001
Creatinine (mg/dl)	1.2 ± 1.01	1.05 ± 0.98	1.46 ± 1	< 0.001
AST (U/l)	50 ± 51	45 ± 46	57 ± 59	< 0.001
ALT (U/l)	44 ± 49	46 ± 55	39 ± 35	0.319
CRP (mg/l)	103 ± 82	79 ± 71	141 ± 85	< 0.001
LDH (U/l)	636 ± 335	527 ± 234	822 ± 397	< 0.001
D-dimer (ng/ml)	2634 ± 8313	1463 ± 3543	4781 ± 12,922	< 0.001
CPK (U/l)	237 ± 319	174 ± 226	312 ± 391	< 0.001
ASTtoALTratio	1.38 ± 0.68	1.22 ± 0.62	1.68 ± 0.68	< 0.001

Pearson *χ*^2^ test, independent sample *T*-test or Mann–Whitney test was used to compare groups

*CKD* chronic kidney disease, *COPD* chronic obstructive pulmonary disease, *AST* aspartate aminotransferase, *ALT* alanine aminotransferase, *CPR* C-reactive protein, *LDH* lactic dehydrogenase, *CPK* creatine phosphokinase

### Comparison between deceased and discharged patients

In total 142 patients (38.1%) died. As expected, patients who experienced in-hospital death were older (78 vs 68 years, *p* < 0.001) and had a higher prevalence of most comorbidities including CVD (46.4% vs 32.5%, *p* = 0.007) and hypertension (78.1 vs 56.8%, *p* < 0.001), whereas only a trend was found for diabetes (23.2 vs 15.6%, *p* = 0.064) and no significant differences were found in gender distribution. Cough was a more common presenting symptom among discharged patients (45.9% vs 28.2%, *p* = 0.001), whereas dyspnea was more frequently encountered in those who died (85.9% vs 62.3%, *p* < 0.001).

**Fig. 1:**
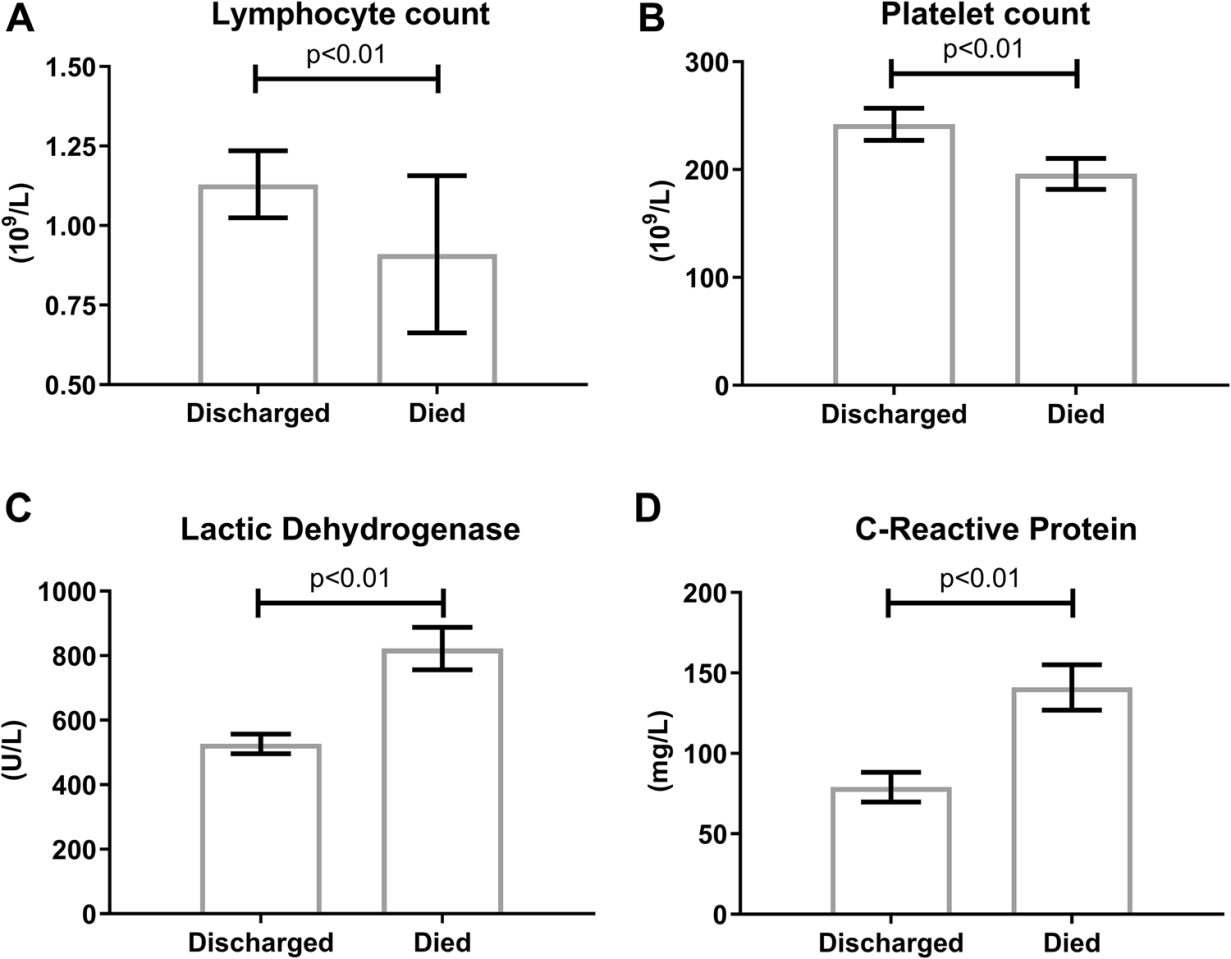
Biochemical features of the study population segregated according to in-hospital death or discharge **a** Lymphocyte count; **b** Platelet count; **c** Lactic dehydrogenase; **d** C-reactive protein

Deceased patients also exhibited less favorable laboratory features at the time of hospital admission, including lower lymphocyte (0.91 vs 1.13 × 10^9^/l, *p* < 0.001) and platelet count (196 vs 242 × 10^9^/l, *p* < 0.001) and higher CRP (141 vs 79 mg/l, *p* < 0.001) and LDH (822 vs 527 U/l) values (Fig. 1).

### Comparison between patients with and without diabetes

Features of the entire population according to diabetes status are shown in Table 2. No significant differences were found between patients with and without diabetes with regards to sex, age (74 vs 71 years, *p* = 0.518) and frequency of symptoms at hospital admission.

**Table 2:** Demographic and laboratory features of patients with diagnosed COVID-19 segregated by T2DM

	No diabetes	Diabetes	*p *value
*N* (female/male)	304 (108/196)	69 (21/48)	0.422
Age (years)	71 ± 14	74 ± 11	0.518
≤ 55 (%)	15.5% (47)	5.8% (4)	0.148
56–75 (%)	36.5% (111)	43.5% (30)	
76–85 (%)	34.5% (105)	39.1% (27)	
> 85 (%)	13.5% (41)	11.6% (8)	
Intensive care unit (%)	14.8% (45)	18.8% (13)	0.403
In-hospital death	35.9% (109)	47.8% (33)	0.064
Comorbidities			
Hypertension (%)	60.9% (182)	82.1% (55)	0.001
CKD (%)	10.5% (32)	23.2% (16)	0.005
Tumors (%)	14.3% (43)	10.1% (7)	0.359
Cardiovascular diseases (%)	35.8% (108)	46.4% (32)	0.101
COPD (%)	10.4% (31)	11.6% (8)	0.765
Symptoms at admission			
Cough (%)	39.8% (121)	36.2% (25)	0.583
Dyspnea (%)	70.7% (215)	73.9% (51)	0.597
Fever (≥ 37.5 °C) (%)	83.2% (253)	79.7% (55)	0.487
Diarrhea (%)	5.3% (16)	2.9% (2)	0.408
Conjunctivitis (%)	0.7% (2)	1.4% (1)	0.506
Laboratory features at admission			
White blood cells (10^9^/l)	7.91 ± 4.11	8.04 ± 3.60	0.130
Lymphocytes (10^9^/l)	1.01 ± 1.08	1.21 ± 1.27	0.217
Neutrophils (10^9^/l)	6.29 ± 3.84	6.51 ± 3.03	0.200
Hemoglobin (g/dl)	12.94 ± 2.06	12.35 ± 1.87	0.040
Platelets (10^9^/l)	228 ± 111	208 ± 84	0.306
Creatinine (mg/dl)	1.18 ± 1.05	1.30 ± 0.79	0.075
AST (U/l)	51 ± 47	44 ± 67	0.004
ALT (U/l)	46 ± 51	33 ± 35	0.008
CRP (mg/l)	104 ± 84	98 ± 74	0.730
LDH (U/l)	621 ± 304	699 ± 439	0.289
D-dimer (ng/ml)	2372 ± 7693	3759 ± 10,601	0.233
CPK (U/l)	253 ± 349	184 ± 183	0.490
AST to ALT ratio	1.36 ± 0.67	1.47 ± 0.71	0.254
Anti-diabetic therapy			
Non-pharmacologic	0.0%	15.9%	–
Oral antidiabetic drugs	0.0%	43.5%	–
Insulin only	0.0%	31.9%	–
Combined therapy	0.0%	8.7%	–

Pearson *χ*^2^ test, independent sample *T*-test or Mann–Whitney test was used to compare groups

*CKD* chronic kidney disease, *COPD* chronic obstructive pulmonary disease, *AST* aspartate aminotransferase, *ALT* alanine aminotransferase, *CPR* C-reactive protein, *LDH* lactic dehydrogenase, *CPK* creatine phosphokinase

As expected both hypertension and CKD were more common among patients with diabetes, but no differences were found in the prevalence of COPD, cancer and CVD. Most laboratory features were not different between groups, except for lower AST, ALT and hemoglobin values and a trend towards a higher creatinine in patients with diabetes.

We found a significant difference in length of stay (LOS) between discharged and deceased patients (16.1 ± 9.7 vs 10.7 ± 11.0 days respectively, *p* < 0.001). However no significant difference in LOS was found between non-diabetic and diabetic patients (14.3 ± 10.8 vs 12.8 ± 9.2 days respectively, *p* = 0.397) even when the analysis was conducted in discharged and deceased patients separately (*p* = 0.934 and *p* = 0.520, respectively).

As far as antidiabetic treatment regimen was concerned 40.6% of patients with diabetes were on insulin, 43.5% on oral antidiabetic drugs and 15.9% were on non-pharmacological treatment. Finally, steroid therapy was not used differently in patients with and without diabetes (11.8% and 13.0% respectively, *p* = 0.782).

In the multivariable logistic regression model (Table 3) older age (Relative Risk [RR] 1.06 [95% confidence interval (CI) 1.04–1.09] per 1 year increase, *p* < 0.001), COPD (RR 1.82 [95% CI 1.13–2.35]), diabetes (RR 1.56 [95% CI 1.06–2.02]), higher levels of CRP (RR 1.70 [95% CI 1.36–2.07] per standard deviation increase, *p* < 0.001) and higher concentrations of LDH (RR 2.14 [95% CI 1.65–2.68] per standard deviation increase, *p* < 0.001) were independently associated with in-hospital mortality, whereas sex, CKD, CVD and hypertension were not. Mortality rate in patients included in the multivariable model was not different from overall mortality (36.2% vs 38%, *p* = 0.22).

**Table 3:** Risk factors for in-hospital mortality

	RR	95% CI	*p* value
Male sex	1.135	0.713–1.623	0.564
Age (per 1 year increase)	1.061	1.035–1.087	< 0.001
Comorbidities			
Diabetes	1.559	1.051–2.028	0.030
Hypertension	0.977	0.419–1.223	0.927
CKD	0.891	0.286–1.311	0.781
Cardiovascular diseases	0.916	0.546–1.396	0.712
COPD	1.821	1.133–2.349	0.019
Laboratory features at admission			
CRP	1.699*	1.364–2.070	< 0.001
LDH	2.137*	1.654–2.679	< 0.001
Platelets	0.787*	0.607–1.012	0.075
Creatinine	1.099*	0.837–1.397	0.562

*Is intended per standard deviation increase

*RR* relative risk, *CI* confidence interval, *COPD* chronic obstructive pulmonary disease, *CKD* chronic kidney disease, *CRP* C reactive protein, *LDH* lactate dehydrogenase

Finally, in a model considering only demographic variables and pre-existing comorbidities, increasing age and male sex were significantly associated with higher mortality (Table 4).

**Table 4:** Role of demographic variables and pre-existing comorbidities on the risk of in-hospital mortality

	RR	95% CI	*p* value
Male sex	1.450	1.084–1.819	0.015
Age (per 1 year increase)	1.065	1.029–1.086	< 0.001
Diabetes	1.228	0.850–1.633	0.253
Hypertension	1.321	0.909–1.806	0.137
CKD	1.127	0.710–1.602	0.580
Cardiovascular diseases	0.844	0.570–1.183	0.348
COPD	1.449	0.944–1.946	0.084

*RR* relative risk, *CI* confidence interval, *CKD* chronic kidney disease, *COPD* chronic obstructive pulmonary disease

## Discussion

In the present study we show that among patients hospitalized for COVID-19 in a single center in Northern Italy, a history of diabetes was associated with an increased mortality risk, which was independent from several confounding factors and comorbidities.

An association between diabetes, glycemic control and COVID-19 outcomes has been previously suggested in several studies, mostly coming from China [15, 16]. It is important to note, however, that clinical features of patients reported in previous studies were different from ours on several aspects and that adjustment for confounding variables was heterogeneous [8, 17]. Finally, different endpoints were considered, going from radiographic findings to ICU admission to in-hospital mortality [7]. Our population was significantly older compared with that of most studies from China with a mean age of ~ 70 years instead of ~ 60. As a consequence, most comorbidities were also more common, including hypertension, diabetes and CVD. Given the high burden of comorbid conditions in our population we made an effort to control for factors that were more likely to influence prognosis and still identified diabetes as a factor that could moderately increase the risk of in-hospital mortality.

It should be noted that our study was performed in a hospital setting including patients managed in the ICU and therefore represents the severe spectrum of COVID-19 affecting frail patients. As a result, while the mean age of our population was significantly higher when compared to Italian data from the Istituto Superiore di Sanità on all infected patients (62 years), the features of deceased patients were similar (median age of 80 years and a prevalence of diabetes of ~ 30%).

Several explanations of the association between diabetes and worse clinical outcome can be proposed [18]. In general, patients with diabetes are more susceptible to a wide range of infections because of alterations in neutrophil chemotaxis, cytokine production and impaired T-cell responses as a consequence of hyperglycemia [19–21]. With this regard a recent non peer-reviewed study clearly showed that diabetic patients experienced a delayed clearance of SARS-CoV-2 [22].

A possible role of antidiabetic therapy cannot be excluded and special interest has been devoted to dipeptidyl-peptidase 4 (DPP4) inhibitors, as DPP4 was one of the receptors involved in the infection from a related coronavirus (Middle East Respiratory Syndrome—Coronavirus). To date, evidence of a possible role of this class of drugs on clinical outcomes is limited to our knowledge to a small study showing a neutral effect [23]. On the same lines sodium-glucose transporter 2 (SGLT2) inhibitors may exert a positive effect in these patients by raising hematocrit, reducing intracellular sodium and calcium and reduce inflammation [24]. A recent study, however, did not show any benefits in terms of length of hospital stay in Italian patients with COVID-19 [25]. Unfortunately, the number of patients with diabetes in our cohort was not large enough to evaluate a possible contribution of specific anti-diabetic drugs.

Finally, patients with diabetes often have comorbid hypertension, heart failure or proteinuria and are frequently treated with blockers of the renin–angiotensin–aldosterone system. Since angiotensin converting enzyme 2 participates in SARS-CoV-2 cell entry process, it was hypothesized that these drugs could increase the risk of infection or worsen the prognosis related to COVID-19. A recent study from the Lombardy region, however, disproved this theory [26].

Some previous studies suggested that a history of hypertension and CVD had a detrimental effect on COVID-19 severity and death. We believe that older age and a more complex set of comorbidities in our patients compared with previous studies may modulate the effect of these conditions on survival. Our data align, on the other hand, with recent results obtained from a multicenter Italian study investigating this issue, which showed that diabetes, but not hypertension or CAD were associated with death [27].

The present study has several limitations that need to be addressed. First, the lack of data on anthropometric parameters in most patients did not allow us to calculate body mass index and to control for the impact of obesity on the primary outcome. Second, we did not have data on glycemic control for most subjects and cannot infer whether any difference in clinical outcomes existed between controlled and uncontrolled diabetes. Third, given that it was performed in the hospital setting and included mostly elderly patients, our results cannot be extended to all individuals with COVID-19. Finally, we were not able to collect data on the fraction of inspired oxygen patients were exposed to, and therefore could not interpret the oxygen saturation at admission, which was associated with progression to death in elderly patients in a recent study [28].

In conclusion, among patients hospitalized with COVID-19 in a single center in northern Italy, risk of in-hospital death was higher in patients with diabetes compared to non-diabetic individuals. Predictors of the primary outcome were increasing age, COPD and higher LDH and CRP values. More intensive surveillance of patients with these conditions may be warranted.

## Data Availability

The datasets are available from the corresponding author on reasonable request.

## Abbreviations

CKD: Chronic kidney disease
COPD: Chronic obstructive pulmonary disease
CVD: Cardiovascular disease
COVID-19: Coronavirus disease 2019
